# Down-regulation of *AUXIN RESPONSE FACTORS 6* and *8* by microRNA 167 leads to floral development defects and female sterility in tomato

**DOI:** 10.1093/jxb/eru141

**Published:** 2014-04-10

**Authors:** Ning Liu, Shan Wu, Jason Van Houten, Ying Wang, Biao Ding, Zhangjun Fei, Thomas H. Clarke, Jason W. Reed, Esther van der Knaap

**Affiliations:** ^1^The Ohio State University, Ohio Agricultural Research and Development Center, Department of Horticulture and Crop Science, Wooster, OH 44691, USA; ^2^The Ohio State University, Department of Molecular Genetics, Columbus, OH 43210, USA; ^3^Boyce Thompson Institute for Plant Research, Ithaca, NY 14853, USA; ^4^University of North Carolina, Department of Biology, Chapel Hill, NC 27599-3280, USA

**Keywords:** ARF6, ARF8, expression, female sterility, flower development, tomato.

## Abstract

Auxin regulates the expression of diverse genes that affect plant growth and development. This regulation requires AUXIN RESPONSE FACTORS (ARFs) that bind to the promoter regions of these genes. *ARF6* and *ARF8* in *Arabidopsis thaliana* are required to promote inflorescence stem elongation and late stages of petal, stamen, and gynoecium development. All seed plants studied thus far have *ARF6* and *ARF8* orthologues as well as the microRNA *miR167*, which targets *ARF6* and *ARF8*. Whether these genes have broadly conserved roles in flower development is not known. To address this question, the effects of down-regulation of *ARF6* and *ARF8* were investigated through transgenic expression of *Arabidopsis MIR167a* in tomato, which diverged from *Arabidopsis* before the radiation of dicotyledonous plants approximately 90–112 million years ago. The transgenic tomato plants overexpressing *MIR167a* exhibited reductions in leaf size and internode length as well as shortened petals, stamens, and styles. More significantly, the transgenic plants were female-sterile as a result of failure of wild-type pollen to germinate on the stigma surface and/or to grow through the style. RNA-Seq analysis identified many genes with significantly altered expression patterns, including those encoding products with functions in ‘transcription regulation’, ‘cell wall’ and ‘lipid metabolism’ categories. Putative orthologues of a subset of these genes were also differentially expressed in *Arabidopsis arf6 arf8* mutant flowers. These results thus suggest that *ARF6* and *ARF8* have conserved roles in controlling growth and development of vegetative and flower organs in dicots.

## Summary

Investigations into the role of tomato ARF6 and ARF8 reveal that they are critical components in floral and gynoecium development before anthesis.

## Introduction

Various hormonal signals regulate flower and subsequent fruit development (Kelley and Gasser, 2009; Nemhauser *et al.*, 1998; Nitsch, 1952; Serrani *et al.*, 2008; Vivian-Smith and Koltunow, 1999). Among them, auxin is critical in regulating gene expression within the flower and young fruit (Dharmasiri *et al.*, 2005; Guilfoyle, 1986; Liscum and Reed, 2002). Auxin response factors (ARFs) bind to auxin response elements (5′-tgtctc-3′ or other similar sequences) in the promoter regions of numerous early auxin-inducible genes (Boer *et al.*, 2014; Franco-Zorrilla *et al.*, 2014; Tiwari *et al.*, 2003; Ulmasov *et al.*, 1997). Typical ARF transcription factors contain three domains: (i) a conserved N-terminal DNA-binding domain (DBD) of the B3 family, (ii) a non-conserved middle region, which may either activate or repress gene expression, and (iii) a conserved C-terminal dimerization domain (CTD) including motif III and motif IV which can dimerize with Aux/IAA (auxin/indoleacetic acid protein) repressors and other ARFs (Guilfoyle and Hagen, 2007; Guilfoyle *et al.*, 1998). Among *Arabidopsis* ARF proteins that mediate auxin-induced gene activation (Tiwari *et al.*, 2003; Ulmasov *et al.*, 1999a), *ARF6* and *ARF8* regulate growth in both vegetative and reproductive tissues. *Arabidopsis arf6* and *arf8* single mutants have mild delays in stem elongation and flower organ growth (Nagpal *et al.*, 2005). However, *arf6 arf8* double mutants have more severe developmental defects, indicating that ARF6 and ARF8 have partially overlapping functions. *Arabidopsis arf6 arf8* flowers arrest as closed buds with short petals, short stamens, and indehiscent anthers, as well as defects in gynoecium growth and support of pollen tube growth (Nagpal *et al.*, 2005). Moreover, ARF6 and ARF8 promote jasmonic acid (JA) production, which in turn induces the expression of *MYB21* and *MYB24* required for petal, stamen, and gynoecium growth at anthesis (Reeves *et al.*, 2012; Tabata *et al.*, 2010). Hence, in *Arabidopsis*, ARF6 and ARF8 coordinate the development of petals and both male and female organs at the transition from closed buds to mature fertile flowers, which contributes to efficient fertilization.

All seed plants seem to have *ARF6* and *ARF8* orthologues (Axtell and Bartel, 2005; Oh *et al.*, 2008; Remington *et al.*, 2004; Xing *et al.*, 2011; Yang *et al.*, 2006), yet their functions have not been studied beyond *Arabidopsis*. Whether these genes have broadly conserved roles in flower development thus remains an outstanding question. The putative importance of *ARF6* and *ARF8* in plant development is suggested by the observation that they are probably targets of *microRNA167* (*miR167*) in all analysed plants (Axtell and Bartel, 2005; Oh *et al.*, 2008; Remington *et al.*, 2004; Xing *et al.*, 2011; Yang *et al.*, 2006). It has been experimentally determined in *Arabidopsis* that *miRNA167* regulates expression of *ARF6* and *ARF8* and that overexpression of the *miR167* precursor gene *MIR167a* phenocopies the *arf6 arf8* double mutant (Ru *et al.*, 2006; Wu *et al.*, 2006).

Tomato provides an excellent model to test the potentially conserved role of ARF6 and ARF8 in flower development as well as their regulation by *miR167*. Tomato and *Arabidopsis* diverged before the radiation of dicotyledonous plants, approximately 90–112 million years ago as estimated by genome sequence comparison and fossil evidence (Ku *et al.*, 2000; Yang *et al.*, 1999). Cultivated tomato (*Solanum lycopersicum*) evolved from *Solanum pimpinellifolium* during the process of crop domestication (Blanca *et al.*, 2012) and they exhibit nearly identical gene sequences (Tomato Genome Consortium, 2012). In our laboratory, we routinely use the *S. pimpinellifolium* accession LA1589 for analyses of tomato development (e.g. Wu *et al.*, 2011; Xiao *et al.*, 2008; Xiao *et al.*, 2009)). We report here that overexpression of the *AtMIR167a* gene in the wild tomato accession LA1589 leads to down-regulation of *SpARF6* and *SpARF8*, which in turn alters flower maturation as in *Arabidopsis*. Our results thus suggest that *ARF6* and *ARF8* play a critical and highly conserved role in flower development in dicot plants.

## Materials and methods

### Plant materials

*S. pimpinellifolium* accession LA1589 was transformed with plasmid *pB7WG2-MIR167a* (Wu *et al.*, 2006) at the Plant Transformation Facility at University of California Davis, USA. Transgenic and wild-type control LA1589 were grown in a greenhouse under standard conditions including supplemental lighting in 5-l pots. The T_0_ transgenic lines were named 092026-001 through 092026-006, 092370-001 and 092370-002. Owing to their female sterility, the severe lines were maintained by backcrossing the pollen to wild-type LA1589. A subset of those backcrossed lines were sowed as pedigree 10S222 (two seedlings: *MIR167a*_222-2 and *MIR167a*_222-3) and were derived from a backcross with T_0_ plant 092026-003 as the pollen donor. For each seedling, four cuttings were rooted, and floral buds from the rooted cuttings were taken for the RNA-seq analysis.

### Identification of *ARF6*, *ARF8*, and *MIR167* genomic sequences from tomato

By using the DNA-binding domain of *Arabidopsis* ARF6 and 8 proteins (defined as amino acids 1 to ~350 by Ulmasov *et al.*, 1999b) as query sequences, four cultivated tomato genes were identified that share a likely common ancestor: *SlARF6A* (Solyc00g196060), *SlARF6B* (solyc07g043610/043620), *SlARF8A* (Solyc03g031970), and *SlARF8B* (Solyc02g037530). As some of the *SlARFs* were not correctly annotated in the tomato genome sequence, we used the validated ORF sequences obtained from Mohamed Zouine and Mondher Bouzayen (University of Toulouse, France) for phylogenetic analyses and the RNA seq analyses shown in Supplementary Table S2 available at *JXB* online. *SlARF6B* and *SpARF6B* are probably pseudogenes in both cultivated and wild tomato, and were excluded from most of the analyses. For *MIR167*, we used the mature microRNA sequence as a query to search for possible *MIR167* gene candidates in the tomato genome (http://solgenomics.net) with Tomato WGS Scaffolds (SL2.40) and an expected threshold value of 1e–0 to avoid false positives. The SoMART software (http://somart.ist.berkeley.edu) was employed to validate *miR167*-mediated regulation of *SlARF6A, SlARF8A*, and *SlARF8B*. The SoMART software uses the small RNA (from http://smallrna.udel.edu) and degradome RNA deep sequencing data (Li *et al.*, 2012). We used the Slicer Detector (SLY1-3 libraries), dRNA mapper (D51Wt library), and SMART COMPARE programs in SoMART with default settings. Both small RNA and degradome RNA deep sequencing data were generated from the tomato cultivar VF36, whereas the genome sequence is that of Heinz1706 cultivar.

### Construction of the class II ARF phylogenetic tree

ARF proteins have a highly conserved DNA-binding domain at the N-terminal region and motif III-IV (two motifs shared with Aux/IAA proteins) at the C-terminal end, but a divergent middle region that generally cannot be aligned among different ARF proteins. The class II ARFs, to which tomato ARF6 and ARF8 belong, are recognized by a glutamine-rich middle region. To generate the phylogenetic tree, we used the core ARF DNA-binding domain that encompasses a span of ~350 amino acids at the N-terminus but excludes the first ~50 variable amino acids (Ulmasov *et al.*, 1999b). *Arabidopsis* ARF protein sequences were obtained from TAIR (The *Arabidopsis* Information Resource, http://www.Arabidopsis.org/). The 8 sequences were aligned using MUSCLE (Edgar, 2004) and a tree was generated using RAxML 7.4.2 (Stamatakis, 2006), both under default settings. To root the tree, the ARF1 DNA binding domain sequence was used as an outgroup. To find the consensus sites for ARF alignment, trimAl (Capella-Gutierrez *et al.*, 2009) was run on the full MUSCLE alignment under strictplus. The trees were visualized using FigTree v 1.3.1 (http://tree.bio.ed.ac.uk/software/figtree/).

### Plant phenotype analyses

For floral organ measurement, flowers were collected from inflorescences of wild-type and transgenic LA1589 plants. The floral organs were separated and placed on half strength MS solid medium for imaging under a dissection microscope (Leica MZFLIII, Germany). Length measurements were performed using Image J (http://rsb.info.nih.gov/ij/). For leaflet measurements, 10 mature terminal leaflets were collected from each line, scanned on a flat-bed scanner and analysed by Tomato Analyzer application (http://www.oardc.ohio-state.edu/vanderknaap/tomato_analyzer.php). The leaf shape index refers to the ratio of height to width.

For light microscopy, samples were fixed in a mixture of 3% glutaraldehyde/4% paraformaldehyde/0.05% Triton X-100 in 0.1M potassium phosphate buffer (pH 7.2) for 2h at room temperature and then overnight at 4°C. After three washes with the potassium phosphate buffer, the samples were dehydrated in a graded ethanol series (25%, 50%, 70% and twice 90%), infiltrated with the EMbed 812 resin (Electron Microscopy Services, Hatfield, PA) and 90% ethanol series (1:3, 1:1, 3:1, twice 100% resin), embedded in airtight gelatine capsules (Electron Microscopy Services) and polymerized overnight at 60°C. Five-µm thick sections were collected on glass slides and stained with 0.5% toluidine blue in 0.1% sodium bicarbonate/25% ethanol for light microscopic observation.

For scanning electron microscopy, samples were infiltrated with 3% gluteraldehyde/2% paraformaldehyde in 0.1M potassium phosphate buffer, pH 7.4, for 2h and subsequently stored in 70% ethanol overnight at 4 °C. The samples were then dehydrated in an ethanol series (25%, 50%, 80%, and twice 95%), critical-point dried, sputter-coated with gold, and viewed at 20kV in an scanning electron microscope (Hitachi S-4700, Japan).

For the hormone experiments, selected floral buds were tagged before the petals in the *MIR167*- transgenic lines turned white, which corresponded to 1–2 days before anthesis in wild-type plants. They were sprayed with 10 µM IAA, 500 µM JA, or 100 µM GA3 (gibberellin A3; in 10% methanol and 0.05% Tween 20) or with buffer alone every 2 d. Fruit development was evaluated one week after the start of hormone treatment.

### Pollen tube staining

Flower buds of LA1589 were emasculated one day before anthesis and pollinated using *MIR167*- transgenic pollen. In parallel, LA1589 pollen was used to pollinate *MIR167*-transgenic flowers. Pollinated pistils were collected after 16h, and kept in a fixative (3:1 of 95% ethanol: glacial acetic acid) overnight. The pistils were cleared with 5M NaOH softening solution for 24h followed by 6-h staining with aniline blue (ABF: 4′4-[carbonyl bis (benzene-4,1-diyl)-bis (imino)]-bisbenzensulfonic acid) in 0.1M K_2_HPO_4_ (pH 10) for callose staining of the pollen tube walls. Slide-mounted pistils were examined using an epi-fluorescence microscope (Leica DM IRB, Germany) equipped with a digital camera. Images were captured at ×50 magnification with QImaging Retiga 2000.

### *In situ* hybridization

To generate RNA probes for RNA *in situ* hybridization of LA1589 tissues, we amplified linear templates for *SpARF6A* and *SpARF8B* from cDNAs using the following specific primer pairs: *SpARF6A*, 5′-TTTCATGAACCGGAACCATT-3′ and 5′-CAAAATTGCCAACGAGTGTG-3′; *SpARF8B*, 5′-GGGAAAG GAAGAGGCTGAAT-3′ and 5′-CGAAAGCTAAAGAAGCCAG GT-3′. An antisense or sense RNA probe was prepared by adding a sequence containing the T7 promoter sequence to the 5′ of the reverse or forward primers, respectively. Probes were labelled by *in vitro* transcription with T7 polymerase using a DIG RNA labeling kit (Roche, Indianapolis, IN, USA). Flower buds at 9 and 4 d before anthesis were fixed with 4% paraformaldehyde in 0.1M phosphate buffer (pH 7.4) and embedded in paraffin. Ten-µm thick sections were obtained with a microtome (American Optical Spencer 820, USA). After dewaxing the sections were washed with 0.2×saline-sodium citrate buffer and incubated with blocking solution (Boehringer, USA) before hybridization with DIG-labelled RNA probes overnight at 55 °C. After buffer washes the DIG-labelled RNA probes were detected by an alkaline-phosphatase-conjugated antibody (Anti-digoxigenin-AP Fab fragments, Roche, USA). After further buffer washes the sections were incubated with the NBT/BCIP solution (Roche, USA) for colour reaction. The mounted slides were observed under the epi-fluorescence microscope (Leica DM IRB) equipped with a digital camera (Q Imaging Retiga 2000, USA).

### RNA isolation and RNA seq library construction

Tomato organs and tissues were collected between 9.00h and 10.00h (supplemental lighting was turned on at 6.00h) and immediately frozen in liquid nitrogen. This time point of collection (short duration and 3h after the lights were turned on) reduced the chance to identify differentially expressed genes due to the circadian clock. Sample collections were performed on separate days for the replicates. Total RNA was extracted with Trizol (Invitrogen Inc. Carlsbad, CA, USA) as described by the manufacturer. RNA quantity and quality were assessed using a Qubit 2.0 fluorometer RNA Assay Kit (Invitrogen Inc. USA) and an Agilent 2100 Bioanalyzer RNA 6000 Nano kit (Agilent, Santa Clara, CA, USA).

The expression analysis for *SpARF6A*, *SpARF8A*, *SpARF8B*, and *STYLE2.1* in LA1589 included the following samples that were collected from greenhouse-grown plants: newly developing leaves approximately 5mm long, full size green terminal leaflets, flower/inflorescence meristems and flower buds up to 10 d before anthesis, flowers at anthesis, 10 d post-anthesis (DPA) fruit, 20 DPA fruit, and breaker stage ripening fruit. The following organs were collected from 7-day-old seedlings grown in petri dishes in a Conviron incubator (Winnipeg, Manitoba, Canada) (16h light and 8h dark): whole root, hypocotyl from below the cotyledons to above the root zone, cotyledons, and vegetative shoot apex containing the vegetative meristem and leaf primordia. Gene expression analysis of wild-type and *MIR167a*- overexpressing LA1589 were evaluated in flower/inflorescence meristems and flower buds up to 10 d before anthesis. This corresponds to the ovule initiation step in floral development (Xiao *et al.*, 2009).

Strand-specific single-end RNA-seq libraries with insert size of approximately 250bp were prepared using the protocol described by Zhong *et al.* (2011) using 10 µg of total RNA. Eight libraries with compatible barcodes were pooled and run on a single lane in a flowcell on the Illumina HiSeq2000 at the Genomics Resources Core Facility at Weill Cornell Medical College (New York, NY, USA) and sequences of 44–51bp length were generated.

### Alignment and analysis of Illumina reads

After the Illumina reads were quality checked, demultiplexed and trimmed, they were clustered per library. The reads were aligned to ribosomal RNA sequences using Bowtie2 (Langmead and Salzberg, 2012) with the ‘very-sensitive-local’ preset parameters to identify and remove the ribosomal RNA sequences from the dataset. Owing to the near-identical gene sequence of the *S. pimpinellifolium* with that of *S. lycopersicum*, the filtered reads were aligned with TopHat2 (Kim *et al.*, 2013) against the cultivated tomato genome allowing for maximum intron lengths of 5000bp, segment lengths of 22bp, and one mismatch per segment. All other parameters were set to the default values. Reads that mapped to up to 20 genes were counted as one for each match. Reads that mapped to more than 20 genes were not counted. Aligned sequences were then separated into sense and antisense, and the counts of aligned reads for each tomato gene model and from each sample were derived using an in-house Perl script. This script also counted reads that partially mapped to the UTRs. Read counts were used to find differentially expressed genes between LA1589 and the two backcrossed progenies miR167a_222-2, and miR167a_222-3 with DESeq2 (Anders *et al.*, 2013) using both parametric and local dispersion fits. Heatmaps and principle components analyses were used to identify possible outlier datasets. Differentially expressed genes with an adjusted *P*<0.05 found in both comparisons were considered for further analysis. Reads per kilobase of exon model per million mapped reads (RPKM) were calculated using an in-house script based on the ITAG 2.3 exon lengths and the total number of reads that mapped to the tomato genome. For the expression analyses of selected genes from different tissues, the average RPKM values for each tissue type are shown. All raw reads for the wild type compared with miR167a_222-2, and miR167a_222-3 lines are deposited in the NCBI sequence read archive (SRA) with accession number SRA057458. All raw reads for expression analysis of the different tissues in LA1589 are deposited with accession number SRA061767. The average RPKM values per sample and for all genes are deposited at http://ted.bti.cornell.edu/cgi-bin/TFGD/digital/home.cgi. Functional category enrichment analysis was performed using the ‘phyper’ function in R (http://www.r-project.org/). Genes were classified into 136 categories based on MapMan BINs using Slyc_ITAG2.3 annotation as the reference.

### AuxRE promoter analysis

The upstream sequence for each significant differentially expressed tomato gene was obtained using custom perl scripts. We used the first 1kb that was 5′ of the start codon or the full 5′ non-coding sequence if the next gene was within 1kb. The sequence and the start codon locations were obtained from http://solgenomics.net/, ITAG v 2.4 for the gene models and v 2.5 for the chromosome sequences. A custom perl script was written to count the instances of two putative AuxRE elements within 20bp of each other on either strand. Three potential AuxRE sequences were analysed: TGTCGG, TGTCGA, and TGTCTC (Franco-Zorrilla *et al.*, 2014), both for pairs of the same elements and for combinations of two different elements, with 1bp mismatches to the search sequences allowed. To evaluate AuxRE occurrence in randomized promoter sequences, the upstream sequences were shuffled 100 times using shuffleseq (http://emboss.sourceforge.net/) and AuxRE pairs were counted each time. The mean, standard deviation, and fraction of the times the shuffled sequence had fewer hits than the actual sequence was reported. We considered AuxRE elements that occurred more in the actual than reshuffled sequences at 0.95 or higher to be significant.

### Northern blot analysis

For Northern blots, 20 µg of total RNA per sample was separated on a 1.2% agarose gel in 1× MOPS buffer and transferred to Hybond N membrane (GE Biosciences, Pittsburgh, PA, USA). Primers used for generating template for labelling were: *SpARF6A*, 5′-AGTGGGTGGCGAGTATCCCG-3′ and 5′-CACCAAGGAGGAGAACATCA-3′; *SpARF8A/B*, 5′-TTTCTC ACAGACACCACCCT-3′; and 5′-CTGCCGTTGACTCATCCC-3′; *SpARF3*, 5′-GATTGTTTTGCTCCCTTGGA-3′ and 5′-TGCTCA GCTGCATCTTCTGT-3′; *STYLE2.1*, 5′-GATTCGCAATCGTCG CTCTA-3′ and 5′-CTGATGATTGCTGCTTCTGG-3′; *eIF4a*, 5′-CA GCTTTTGCCACCAAAAAT-3′ and 5′-TCTGATCCATGTCTCC GTGA-3′. DNA probes were generated using α^32^P-dCTP and a three-cycle PCR amplification of the templates obtained from RT-PCR. Hybridization was performed at 65 °C in modified Church buffer (0.5M sodium phosphate pH 7.2, 1mM EDTA, and 7% SDS). Blots were washed twice with 2×SSC, 0.1% SDS at 65 °C and twice with 0.2×SSC, 0.1% SDS at 65 °C. Radioactive bands were visualized using a Phosphor Imager (Model Storm840, Molecular Dynamics, GE Biosciences, Pittsburgh PA, USA).

For low molecular weight RNA, 25 µg of total RNA extracted from anthesis-stage flowers was suspended in 20 µl of RNA loading buffer (95% formamide, 5mM EDTA, 0.025% SDS, 0.025% bromophenol blue and xylene cyanol FF) and separated in 15% denaturing polyacrylamide gel containing 8M urea. Antisense miR167 (5′-TAGATCATGCTGGCAGCTTCA-3′) and miR166 (5′-GGGGAATGAAGCCTGGTCCGA-3′) probes were prepared by end-labelling with T4-polynucleotide kinase (New England Biolabs, Ipswich MA, USA) in the presence of γ^32^P-ATP. Hybridization was performed at 42 °C in ULTRAhyb ® hybridization buffer (Invitrogen, USA) at 42 °C. Blots were washed with 2×SSC, 0.1% SDS at 42 °C and with 0.2×SSC, 0.1% SDS at 42 °C. Radioactive signals were visualized as mentioned above.

### Southern blot analysis

Genomic DNA was extracted from the young leaves of four-week old tomato plants. Ten µg of genomic DNA was digested with *EcoR*I and *EcoR*V (New England Biolabs, USA), separated on a 0.8% agarose gel, and blotted onto a Hybond-N^+^ membrane (GE Biosciences, USA) under alkaline conditions. The coding region of *BAR* gene amplified with primers (5′-TGCCAGTTCCCGTGCTT-3′ and 5′-CAACTCGATCGAGGGGATC-3′) was used as template for the three-cycle PCR labelling reaction. Hybridization and visualization were performed as described for the northern blot analysis.

## Results

### Identification of tomato *ARF6A/B* and *ARF8A/B*

**Fig. 1.**
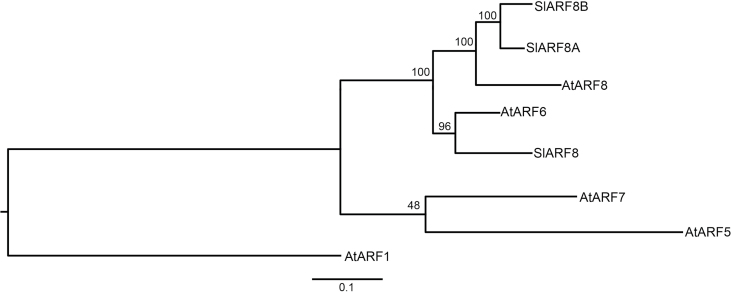
Phylogeny of SlARF6A, SlARF8A and SlARF8B and closely related *Arabidopsis* ARF proteins. The DNA binding motif was used to construct the phylogenetic tree using MUSCLE and RAxML version 7.4.2. AtARF1 is used as outgroup.

The DNA-binding domains of *Arabidopsis* ARF6 and ARF8 were used to identify tomato orthologues from the genome sequence (Tomato Genome Consortium, 2012; Ulmasov *et al.*, 1999b). We found two putative orthologues of *ARF6* (*SlARF6A* Solyc00g196060 and *SlARF6B* Solyc07g043610/043620) and two orthologues of *ARF8* (*SlARF8A* Solyc03g031970 and *SlARF8B* Solyc02g037530). *SlARF6B* is probably a pseudogene given its annotation as two genes (Solyc07g043610 and Solyc07g043620) and given the presence of a premature stop codon in both the wild (LA1589) and cultivated tomato (Heinz1706) sequences. The sequences of *SlARF6A* and *SlARF8A/B* were validated by Mohamed Zouine and Mondher Bouzayen (University of Toulouse, France). These *SlARF*s are clustered in the Class II subclade based on phylogenetic analysis (Fig. 1).

### Expression of *SpARF6A*, *SpARF8A*, and *SpARF8B* in tomato organs and tissues.

**Fig. 2.**
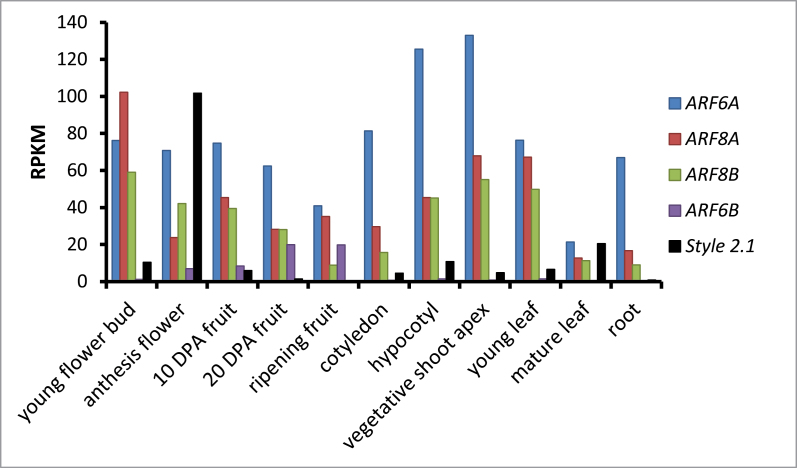
Expression of *SpARF6A*, *SpARF6B*, *SpARF8A*, *SpARF8B* and *STYLE 2.1* in tomato organs and tissues. The values represent average reads per kilobase of gene and per million reads that map to the annotated genome. (This figure is available in colour at *JXB* online.)

**Fig. 3.**
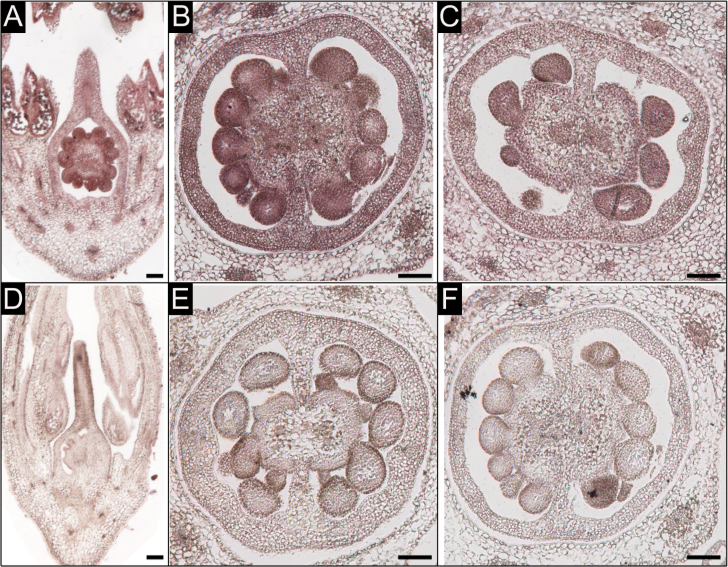
*In situ* hybridization of *SpARF6A* and *SpARF8B* in tomato flowers. (A, B) Wild-type LA1589 flower at 9 and 4 days before anthesis hybridized with the antisense *SpARF6A* probe. (C) Wild-type ovary at 4 days before anthesis hybridized with the antisense *SpARF8B* probe. (D, E) *MIR167* transgenic flowers at 9 and 4 days before anthesis hybridized with the antisense *SpARF6A* probe. (F) A 4-days before anthesis wild-type ovary hybridized with the sense *SpARF6A* probe as control. Bar=100 µm.

The expression of the three *ARF* genes in different organs/tissues of LA1589 was investigated from a dataset that was generated previously (Huang *et al.*, 2013). The gene expression levels were averaged over three-to-four biological replicates and expressed in reads per kilobase per million mapped reads (RPKM) (Supplementary Table S1 available at *JXB* online). In general, *SpARF6A* was expressed at higher levels than *SpARF8A* and *SpARF8B* (Fig. 2). All three genes were expressed in multiple tissues including seedlings, shoot meristems, young leaves, flowers, fruits at multiple stages, and roots. The lowest expression level of the three *ARF* genes was found in mature terminal leaflets. We also evaluated the expression of the *SpARF6B* pseudogene by adding the RPKM for Solyc07g043610 and Solyc07g043620 divided by 2. The gene had a very low expression level except in 20 DPA fruits as well as ripening fruits. The significance of the low but detectable expression level of *SpARF6B* is not clear. *In situ* hybridization of flower buds at 9 and 4 days before anthesis using *SpARF6A* and *SpARF8B* probes demonstrated strong expression levels in the developing ovules and pollen (Fig. 3). This expression pattern suggests potential regulatory roles of these genes in flower development, particularly in the female organs.

### Identification of *MIR167* in tomato

The *Arabidopsis miR167* regulates the expression of *AtARF6* and *AtARF8*. We wanted to know whether this regulatory mechanism is conserved in tomato. Cultivated tomato *miR167* was initially identified in a conventional small RNA cloning approach, and its expression pattern was developmentally regulated based on small RNA gel blot (Itaya *et al.*, 2008) and deep sequencing (http://smallrna.udel.edu). Mature *Sly-miR167* shares an identical sequence with the *Arabidopsis miR167*. Previously, tomato *miR167*-mediated cleavage of *SlARF8B* was confirmed by 5′-RACE (Moxon *et al.*, 2008). The target site is shown in Supplementary Figure S1A available at *JXB* online. We employed the newly developed online software tool SoMART and available small RNA sequencing data from VF36 and Microtom (Li *et al.*, 2012) to evaluate whether *SlARF6A* and *SlARF8A* were also targets of *miR167*. *miR167*-mediated cleavage products were found for *SlARF6A*, *SlARF8A*, and *SlARF8B* (Supplementary Fig. S1A available at *JXB* online). The results indicated that these tomato genes are targets of *miR167*, as are the orthologous *ARF6* and *ARF8* genes in *Arabidopsis*.

The tomato genome contains four putative precursor genes that could encode *miR167* even though only one, *Sly-MIR167,* is registered in miRBase (Griffiths-Jones, 2004; Tomato Genome Consortium, 2012). The genome sequences surrounding these four putative genes share sequence homology to *Ath-MIR167a* and *Sly-MIR167*. Together, this suggests the presence of four *MIR167* genes encoding *miR167* precursors (Supplementary Fig. S1B available at *JXB* online). Thus, we renamed the registered *Sly-MIR16*7 as *Sly-MIR167a-1* and the additional three genes as *Sly-MIR167a-2*, *Sly-MIR167a-3*, and *Sly-MIR167a-4*. The predicted precursor structures of these four *Sly-MIR167* genes are shown in Supplementary Figure S1B available at *JXB* online. We also found the corresponding miR167* sequences (from the other strand of the stem-loop precursors) for these four genes in the small RNA deep sequencing database (http://smallrna.udel.edu). The tomato genome has two additional sequences with high similarity to mature *miR167*. However, we were unable to validate these as *bona fide MIR167* genes based on the absence of a strongly supported predicted stem-loop precursor structure using *in silico* folding analysis (Supplementary Fig. S1C available at *JXB* online), extremely low reads in small RNA deep sequencing (fewer than 10 reads), and the lack of miRNA* sequences for these candidate genes in deep sequencing data (http://smallrna.udel.edu/). Thus, we concluded that tomato has four *MIR167* genes that produce *miR167*, which regulates *SlARF6* and *SlARF8* genes. Thus, regulation of *ARF6* and *ARF8* by the *miR167* family is conserved between *Solanaceae* and *Brassicaceae*.

### Expression of *Arabidopsis MIR167a* in tomato plants decreases expression of *ARF6A* and *ARF8A/B* and causes growth defects.

**Fig. 4.**
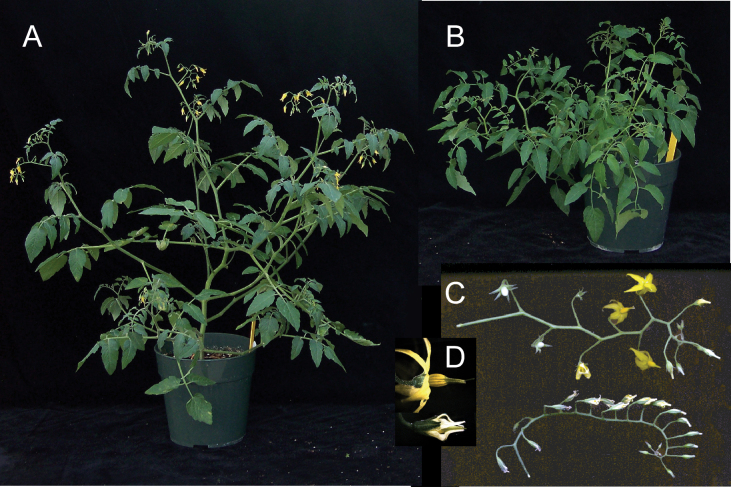
Phenotypes of the *MIR167* transgenic tomato plants. (A) Wild-type LA1589. (B) *MIR167* transgenic tomato line 092026-003 showing reduced plant height/size compared with wild-type control tomato. Photos were taken two months after sowing. (C) Wild-type inflorescence with opened flowers and young fruit (top) and *MIR167* inflorescence with unopened flowers and no fruit (bottom). (D) Close-up mature flowers of wild type (top) and *MIR167* (bottom).

**Fig. 5.**
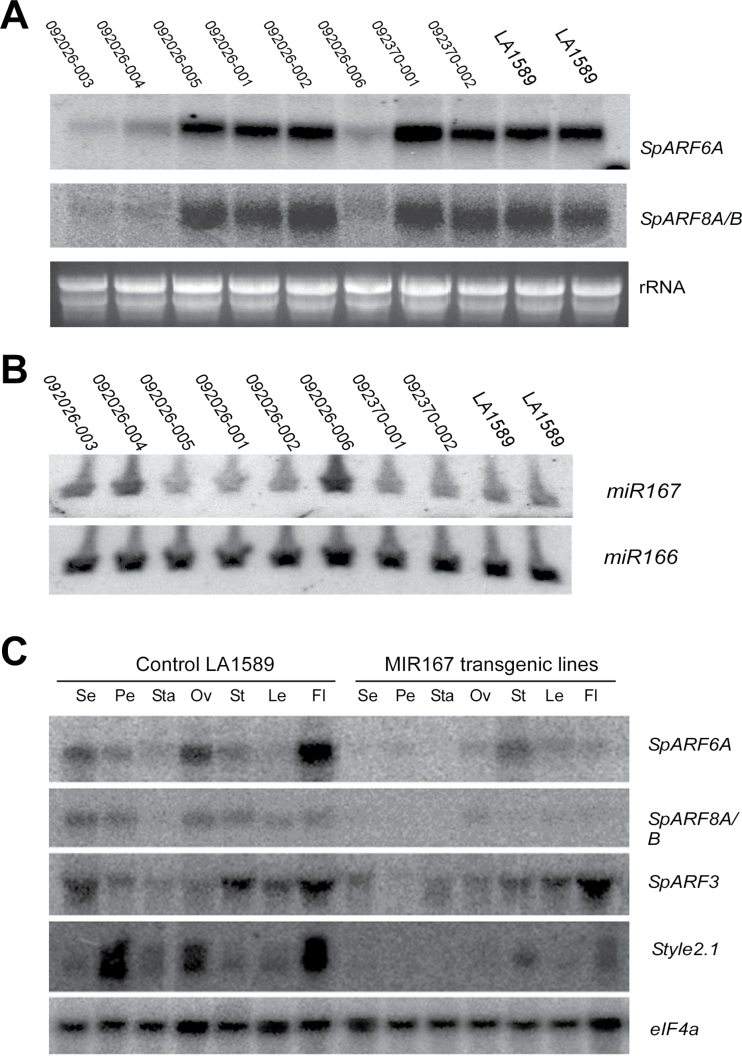
Gene expression analysis in *MIR167* transgenic and wild-type tomato flower and floral organs. (A) Expression of *SpARF6A*, *SpARF8A/B* in anthesis-stage flowers of wild-type, and *MIR167* transgenic lines. (B) Mature *miR167* levels in wild-type and transgenic lines. *miR166* expression served as control. (C) Expression analysis of selected *SpARF* and *Style2.1* in floral organs, terminal leaflets, and whole flowers. Se, sepal; Pe, petal; Sta, stamen; Ov, ovary; St, stem; Le, terminal leaflet; Fl, whole flower at anthesis. Northern blots were carried out with 20 µg total RNA.

We next investigated whether *SpARF6* and *SpARF8* might regulate vegetative and flower development similarly in tomato and in *Arabidopsis*. As *miR167* regulates *ARF6* and *ARF8* genes in both species, we expected that plasmid *pB7WG2-MIR167a*, which expresses *Arabidopsis MIR167a* under the control of the CaMV 35S promoter (Wu *et al.*, 2006), would target tomato *SpARF6* and *SpARF8* genes. We obtained eight independent transgenic LA1589 lines carrying *pB7WG2-MIR167a*. Compared with control lines, three *MIR167a*-transgenic lines (092026-003, 092026-004, and 092026-006) exhibited smaller stature, smaller terminal leaflets, and incomplete floral development (Fig. 4, Table 1). The remaining transgenic lines resembled non-transformed control plants. Southern blot analysis showed that most lines harboured two or more copies of the transgene (Supplementary Fig. S2 available at *JXB* online). However, transgene copy number did not seem to be correlated with the severity of phenotypes. Northern blot analysis of RNA isolated from anthesis-stage flowers showed that the lines with strong phenotypes had a significant reduction in the expression of *SpARF6A* and *SpARF8A/B* (Fig. 5A) as well as increased *miR167* expression over endogenous levels (Fig. 5B). Moreover, RNA seq analysis of very young flower buds in control and transgenic *MIR167* lines showed that *SpARF6A* and *SpARF8B* were most significantly down-regulated (3 and 1.7-fold respectively), whereas *SpARF8A* was not down-regulated in these tissues (Supplementary Table S2 available at *JXB* online). Expression of the pseudogene *SlARF6B* was practically undetectable in very young flower buds (Supplementary Table S2 available at *JXB* online). Therefore, the data demonstrated that increased *miR167* expression in the transgenic tomato plants led to reduced expression of *ARF6* and *ARF8* resulting in altered developmental phenotypes.

**Table 1.** Terminal leaflet characteristics of LA1589 control and *MIR167* transgenic plants

	Wild type	*MIR167* transgenic lines					
	LA1589 (*n*=10)	092026-001 (*n*=10)	092026-002 (*n*=10)	092026-003 (*n*=10)	092026-004 (*n*=10)	092026-005 (*n*=10)	092026-006 (*n*=10)
Perimeter (cm)	15.2±1.6	16.7±1.7	14.7±0.5	10.5±1.2*	11.7±2.2*	14.1±0.6	11.6±1.4*
Area (cm^2^)	7.7±1.2	8.4±0.9	6.4±1.4	3.7±1.1*	3.3±0.4*	5.9±0.7	4.7±0.7*
Maximum width (cm)	2.4±0.3	2.4±0.2	2.2±0.3	1.6±0.2*	1.5±0.1*	2.2±0.2	2.0±0.2*
Maximum length (cm)	5.8±0.5	6.6±0.9	5.6±0.2	4.0±0.4*	4.4±0.4*	5.4±0.3	4.6±0.8*
Leaf shape index	2.4±0.2	2.7±0.4	2.5±0.4	2.5±0.3	2.8±0.2	2.5±0.2	2.3±0.5
Proximal angle	56.2±4.7	52.9±5.4	55.0±3.6	56.5±8.5	58.2±7.4	52.5±6.4	57.2±6.3

The values represent the mean±SD for wild type and primary transformants. *Denotes significant difference by student’s t test (*P*<0.05) compared with wild type.

The three *MIR167a*-transgenic tomato lines that showed reduced *ARF6* and *ARF8* expression and severe phenotypes were further investigated. The shorter stature of *MIR167a*-plants was caused by reduced internode growth (Table 2). After transition to flowering, control LA1589 carried inflorescences after every third internode as is customary for indeterminate tomato. The internode that carried the first inflorescence was the 7th or 8th internode in both wild type and transgenic lines. In contrast, five of the *MIR167a*-plants carried the second inflorescence after four internodes whereas the other five carried the second inflorescence after three internodes (Table 2), suggesting that down-regulation of *SpARF6* and *SpARF8* delayed inflorescence meristem termination in the sympodial shoot. In addition, floral development in the *MIR167a*-plants differed from wild-type development starting just before anthesis when the flowers failed to open completely as compared with the wild-type controls (Fig. 4C, D). In mature post-anthesis flowers, petals, stamens, and style were all shorter in *MIR167a* plants than in wild-type plants, with the largest defects in petals (Table 3). This reduced organ length was not rescued by exogenous application of MeJA (methyl jasmonic acid), and was partially rescued by application of GA (gibberellic acid; for the style and stamen) (Table 3 and Supplementary Table S3A available at *JXB* online).

**Table 2.** Internode lengths in wild-type LA1589 and *MIR167* tomato plants

	Internode number									
	1	2	3	4	5	6	7+8^a^	9	10	11+12^b^
Wild type (mm) *n*=10	21.4±2.8	21.5±2.5	23.4±2.4	35.9±8.4	46.9±9.6	54.8±11	107.8±9.4	83.9±13	67.7±8.6	91.2±12.1
*MIR167* (mm) *n*=10	16±2.4*	18.6±2.3*	17.8±1.8*	23.5±2.6*	20.5±3.2*	18.5±2.4*	39.0±4.1*	35.5±4.8*	31.2±4.0*	45.6±6.8*

The values represent the mean±SD. Asterisk denotes significant difference by student’s t-test (*P*<0.05) compared with wild type. ^a^Termination of the vegetative meristem in the inflorescence meristem occurs after the 7th or 8th leaf. Values are the sum of the lengths of internodes just above and below the first inflorescence. ^b^In wild type, sympodial growth continues for exactly three internodes until terminating again in an inflorescence meristem. In *MIR167*, the second inflorescence forms either after 3 or 4 internodes from the first inflorescence. Values are the sum of the lengths of internodes just above and below the second inflorescence.

**Table 3.** Floral organ lengths two days post-anthesis

	LA1589 (*n*=16)	*MIR167* (*n*=26)	*MIR167* + MeJA (*n*=21)
Sepal (mm)	4.9±0.3	4.9±0.2	4.7±0.2
Petal (mm)	12.8±1.6	7.9±0.6*	7.9±0.6*
Stamen cone (mm)	8.7±0.4	7.4±0.5*	7.3±0.4*
Ovary (mm)	1.2±0.1	1.1±0.1	1.0±0.2
Style (mm)	8.8±0.2	6.6±0.9*	6.6±1.3*

The values represent the mean±SD. Asterisk denotes significant difference by student’s t-test (*P*<0.05) compared with wild type. No significant differences were found in *MIR167* transgenics treated with or without methyl jasmonate.

Analyses of multiple flower buds from different positions along the inflorescence showed that the styles of transgenic plants had grown less than those of wild-type plants as early as 5–6 days before anthesis, whereas petal and stamen growth defects occurred just before and after anthesis (Table 3 and Supplementary Table S3B available at *JXB* online). Thus, ectopic expression of *MIR167a* affected petal, stamen, and style growth, with the earliest effects on style elongation and later effects on petal and stamen elongation. Petals also seemed not to senesce after flower opening. In control LA1589, lack of fertilization normally leads to flower drop. In the lines overexpressing *MIR167a*, flowers and floral organs remained on the peduncle well after the normal time of flower opening (Fig. 4C).

We also examined the accumulation of *SpARF6A* and *SpARF8A/B* transcripts in different floral organs of wild-type and transgenic lines. Northern blot analysis showed that in wild type the highest levels of *SpARF6A* and *SpARF8A/B* were found in the ovary, sepal, and petal, but these genes were barely expressed in stamens (Fig. 5C). In *MIR167a*-transgenic plants, the transcripts of *SpARF6A* and *SpARF8A/B* were barely detected in any floral organs. As a control, *SpARF3* was expressed at similar levels in wild-type and transgenic plants (Fig. 5C). We also evaluated the expression of *STYLE2.1*. This gene controls stigma exsertion in tomato and its reduced expression leads to shorter styles, thereby facilitating inbreeding and selfing (Chen *et al.*, 2007). In control plants, expression of *STYLE2.1* was robust in flowers at anthesis, and low or undetectable in other organs and tissue types (Fig. 2). *STYLE2.1* was highly expressed in petal, ovary, and entire flowers in wild-type plants. However, in the *MIR167a* transgenic lines, expression of *STYLE2.1* was markedly reduced in floral organs. These results suggest that *STYLE2.1* may function downstream of *ARF6* and *ARF8* and its reduced expression in *MIR167a*-plants contributed to the reduced style growth.

### Defects in female organs in *MIR167a-*overexpressing tomato plants cause sterility

The *MIR167a* plants did not produce fruit, suggesting that they might be sterile. To investigate whether the lack of fecundity arose from male or female sterility, reciprocal crosses were conducted between *MIR167a*-overexpressing and control tomato plants. Pollination of wild-type pistils with *MIR167a* transgenic pollen resulted in high fruit set (*n*=19 pollinations and 80–85% fruit set for T_0_ lines 092026-003 and 092026-004) and viable seed production. In contrast, pistils from the severe *MIR167a* lines 092026-003 and 092026-004 pollinated with wild-type pollen did not yield any fruit (*n*=15 and 17 pollinations for lines 003 and 004, respectively). Thus, the *MIR167a*-transgenic tomato plants were female sterile but male fertile.

To determine the basis for the female sterility of *MIR167a* plants, we examined pollen tube growth in pistils after manual pollination. Whereas *MIR167a* pollen tubes grew well in wild-type pistils, wild-type pollen was unable to grow in the styles or stigmas of the severe *MIR167a* plants (Supplementary Fig. S3A–C available at *JXB* online). To ensure that pollen would stick to the stigmas in these situations, we applied lanolin onto the stigmas before pollination. Regardless of lanolin application, no pollen tubes were found in styles of the severe *MIR167a-*transgenic lines (data not shown). These results suggested that female sterility was due to defects in pollen recognition on the stigma surface, pollen germination and/or pollen tube growth in the transmitting tracts of *MIR167a* plants.

**Fig. 6.**
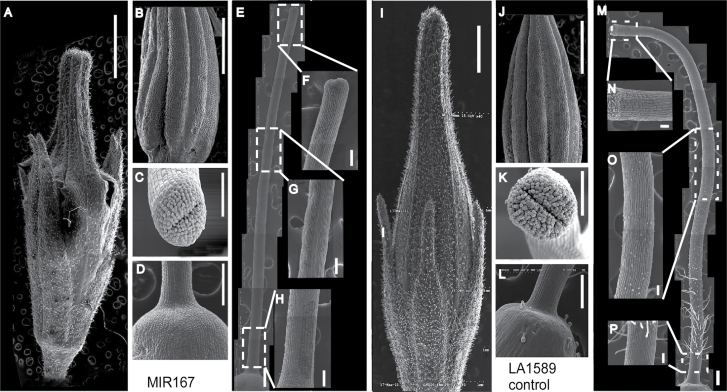
SEM of floral organs from *MIR167* and control tomato plants. Flowers were collected 1 day before anthesis. (A–H) *MIR167* transgenic plants. (I–P) Wild-type LA1589. Whole flower buds just before opening (A and I); stamen (B and J); stigma (C and K); top of ovary and base of style (D and L). Style morphology of the *MIR167* transgenic (E–H) and wild-type (M–P) plants. Close-up of the corresponding style regions boxed in white are shown in (F–H) for *MIR167* and (N–P) for control. Size bar in A, B, I, J =1mm; size bar in C, K=100 µm; size bar in D, E, G, M = 200 µm; size bar in F–H and N–P=500 µm.

To characterize pistil growth in greater detail, we examined wild-type and *MIR167a* tomato floral organs by scanning electron microscopy (SEM). As shown in Fig. 6, overall morphology of the sepals, petals, stamen, and stigma seemed similar in wild-type and *MIR167a* lines although petals, stamens and style were shorter in the transgenic lines as noted above. Notably, trichomes were abundant near the bases of the styles in wild-type plants but were completely absent from the styles of *MIR167a* plants. Jasmonate-insensitive mutants of tomato also lack stylar trichomes (Li *et al.*, 2004). However, application of exogenous MeJA did not restore the trichome defect of *MIR167* styles, and application of IAA, MeJA, or GA did not restore female fertility (data not shown). Cross-sections of style and ovary showed similar cellular organization in wild type and transgenic lines, except possibly for the placenta, which seemed smaller in the *MIR167* plants (Fig. S3D–M). In summary, our results suggest the defect in female fertility probably arose as a result of arrested development at the stigma surface and/or in the transmitting tract. More generally, the stigma or style defects and the lack of trichomes together indicate that the pistils of *MIR167a* plants failed to mature.

To determine the cellular mechanism by which reduced *ARF6* and *ARF8* expression led to reduced style length, the cell number and length of mature styles were measured one day before anthesis in both transgenic and wild-type flowers. Close to the ovary, cell length varied less as compared with that in the distal regions of the style where cell length was three-to-four times longer in wild-type than in *MIR167a* styles. On the other hand, total cell number along the style was higher in *MIR167a* transgenic lines than in wild-type control lines (Table 4). Thus, *MIR167a* overexpression and the concomitant reduction in *SpARF6* and *SpARF8* expression led to shorter styles through a greatly reduced cell length even though cell number increased.

**Table 4.** Cell length and number in the styles of control and *MIR167* flowers

Distance from the ovary	Control LA1589	*MIR167*
Proximal, region 1 (µm)	27.9±5.0	19.5±1.8
Region 2 (µm)	107.4±25.5	58.9±6.5
Region 3 (µm)	247.0±44.1	61.7±6.6
Distal, region 4 (µm)	79.0±19.4	30.6±2.6
Total cell number	51±3.9	84.3±6.0

Region 1 is closest to the ovary. Region 2 and 3 are in the middle, whereas region 4 is furthest away from the ovary, proximal to the stigma. Epidermal cell length and number of the styles were evaluated using SEM.

### RNA-seq analysis of floral tissues of wild-type and *MIR167a*-transgenic lines

To gain molecular insights into the impacts of *miR167a*-regulated *ARF6* and *ARF8* expression on flower development, we used RNA-seq to profile the global expression patterns of tomato genes in very young flower buds of *MIR167a* plants in comparison with those in non-transgenic control plants. The samples were collected at flower developmental stages corresponding to ovule initiation and earlier, so that the primary effects of decreased *SpARF6* and *SpARF8* expression on flower development could be assessed before general growth defects occurred. Statistical evaluation using the DESeq2 package revealed a total of 1094 and 1737 genes that were differentially expressed in miR167a_222-2 and miR167a_222-3 progenies, respectively, as compared with those in wild-type plants. These lines were derived from a backcross of 092026-003 pollen with LA1589. Of these, 687 differentially expressed genes were shared in the two data sets (Supplementary Table S4 available at *JXB* online). Gene ontology (GO) analyses of these 687 genes showed that most of the bin categories containing four or more members were collectively down-regulated in *MIR167a* plants compared with the control, whereas other bins show a mixture of up- and down-regulated genes. No bin category with four or more members was up-regulated in *MIR167a* plants. Many genes involved in transcription regulation were differentially expressed. Of these, MADS box, AP2/EREBP, and MYB-domain/MYB-related genes were represented with the highest numbers. The MADS box genes were mostly up-regulated whereas AP2/EREBP genes were mostly down-regulated in *MIR167a* plants compared with those in wild-type plants (Supplementary Table S4 available at *JXB* online). Many genes encoding proteins in cell wall metabolism were under-expressed in the *MIR167a* flower buds. These included UDP-glucosyl and -glucoronyl transferases, gluco-, manno- and galactosidases, invertases and pectin methyl transferase inhibitors, and beta-1,3-glucan hydrolases. In the hormone bin, genes mostly involved in auxin and ethylene metabolism were differentially expressed, where others were up- or down-regulated. Many genes involved in protein degradation and post-translational modifications were differentially expressed, as were genes encoding receptor kinases and factors in calcium signalling. In the category ‘Development’, we found that genes with similarity to *LFY*, *CUC*s, and *UFO* were up-regulated in *MIR167a* plants, in addition to many others whose roles in development are less clear. Lastly, many genes involved in transport were differentially expressed, in particular those that transport sugars and peptides as well as ABC transporters (Supplementary Table S4 available at *JXB* online). Promoter analyses of the differentially expressed tomato genes showed that 109 out of 686 genes contained pairs of putative AuxRE elements that occurred more than random 95% of the time or more (Supplementary Table S5 available at *JXB* online). The list included two *SAUR* genes, an *expansin* gene, and an *ARF19*-like gene. *Arabidopsis* homologues of these genes are auxin-inducible, suggesting that a subset of all the differentially expressed genes were directly downstream of ARF6 and ARF8.

To determine whether certain functional categories were overrepresented in the list of differentially expressed genes, we performed hypergeometric tests with the genes in each MapMan BIN (Thimm *et al.*, 2004; Table 5). At the same time, we also compared the differentially expressed tomato genes with those identified in a previous *Arabidopsis* microarray study of wild-type and *arf6 arf8* mutant stage 12 flower buds (just before anthesis) (Reeves *et al.*, 2012). This comparison led to the identification of 185 common differentially expressed genes corresponding to the same Phytozome family in tomato and *Arabidopsis*. Of these, 142 were down-regulated and 43 were up-regulated as a result of overexpression of *MIR167a* (Supplementary Table S6 available at *JXB* online). Thus, decreased ARF6 and ARF8 activity affected the expression of a conserved set of genes in tomato and *Arabidopsis*. For example, in the category ‘Regulation of transcription’, ARFs and bZIP transcription factors were overrepresented in the common set, as expected, whereas general transcription factor genes (all were members of the GRF family) were overrepresented in the tomato-specific set only. Some other common genes included those in the ‘Cell wall’, ‘Transport’, and ‘Hormone’ categories. Common genes in the ‘Other’ category included those encoding MAP kinases, *O*-methyl transferases, TCA cycle components, biodegradation machinery, GDSL-motif lipases, tetrapyrrole synthesis enzymes, and UDP-glucosyl and -glucoronyl transferases.

**Table 5.** Functional category enrichment of differentially expressed genes between wild-type LA1589 and *MIR167* transgenic lines

	MapMan BinCode	Category	All^a^		Common^b^		Unique^c^		Genome^d^		*P*-value^f^	*P*-value (common)	*P*-value (unique)
			num^e^	Freq	num	freq	num	freq	num	freq			
Regulation of transcription	27.3.50	General transcription (GRF family)	4 (0)	0.57%	0	0	4 (0)	0.78%	31	0.09%	**0.011**	0.263	**0.010**
	27.3.24	MADS box transcription factor family	7 (6)	0.99%	2 (1)	1.05%	5 (5)	0.97%	101	0.28%	**0.016**	0.060	**0.039**
	27.3.4	Auxin response factor family	3 (2)	0.43%	2 (2)	1.05%	1 (0)	0.19%	26	0.07%	**0.016**	**6.02E–03**	0.138
	27.3.40	Aux/IAA family	3 (3)	0.43%	1 (1)	0.52%	2 (2)	0.39%	25	0.07%	**0.016**	**0.037**	**0.039**
	27.3.26	MYB-related transcription factor family	3 (1)	0.43%	0	0	3 (1)	0.58%	49	0.14%	0.059	0.332	**0.039**
	27.3.21	GRAS transcription factor family	3 (0)	0.43%	0	0	3 (0)	0.58%	53	0.15%	0.069	0.340	**0.047**
	27.3.35	bZIP transcription factor family	4 (3)	0.57%	2 (1)	1.05%	2 (2)	0.39%	82	0.23%	0.071	**0.042**	0.232
Cell wall	10.6	Cell wall degradation	10 (9)	1.42%	3 (3)	1.57%	7 (6)	1.36%	181	0.51%	**0.016**	0.060	**0.039**
	10.8.1	Pectinesterases	6 (6)	0.85%	3 (3)	1.57%	3 (3)	0.58%	87	0.24%	**0.016**	**0.012**	0.111
	10.1	Cell wall precursor synthesis	5 (4)	0.71%	2 (2)	1.05%	3 (2)	0.58%	68	0.19%	**0.019**	**0.030**	0.070
	10.7	Cell wall modification	4 (4)	0.57%	3 (3)	1.57%	1 (1)	0.19%	81	0.23%	0.071	**0.010**	0.437
	10.2	Cellulose synthesis	3 (3)	0.43%	2 (2)	1.05%	1 (1)	0.19%	66	0.19%	0.100	**0.028**	0.354
Transport	34.2	Transport of sugars	5 (2)	0.71%	2 (1)	1.05%	3 (1)	0.58%	80	0.23%	**0.029**	**0.042**	0.091
	34.1	p- and v-ATPases	3 (3)	0.43%	2 (2)	1.05%	1 (1)	0.19%	62	0.17%	0.084	**0.027**	0.334
	34.15	Potassium transport	3 (2)	0.43%	2 (1)	1.05%	1 (1)	0.19%	62	0.17%	0.084	**0.027**	0.334
Lipid metabolism	11.1	Fatty acid synthesis and elongation	7 (6)	0.99%	2 (2)	1.05%	5 (4)	0.97%	132	0.37%	**0.029**	0.100	0.060
	11.8	Lipid metabolism (steroids, squalene etc)	6 (5)	0.85%	0	0	6 (5)	1.17%	114	0.32%	**0.039**	0.503	0.023
Hormone metabolism	17.6	Gibberellin	3 (1)	0.43%	2 (0)	1.05%	1 (1)	0.19%	53	0.15%	0.069	**0.021**	0.289
	17.2.3	Auxin-induced-regulated- responsive-activated	7 (4)	0.99%	4 (2)	2.09%	3 (2)	0.58%	218	0.61%	0.140	**0.032**	0.479
Others	29.7	Protein glycosylation	6 (6)	0.85%	2 (2)	1.05%	4 (4)	0.78%	49	0.14%	**6.6E–03**	**0.021**	**0.016**
	26.22	Short chain dehydrogenase/ reductase	7 (6)	0.99%	1 (1)	0.52%	6 (5)	1.17%	86	0.24%	**0.011**	0.169	**0.011**
	30.6	MAP kinases	3 (3)	0.43%	3 (3)	1.57%	0	0	17	0.05%	**0.011**	**1.24E–04**	0.331
	16.2	Phenylpropanoids metabolism.	10 (3)	1.42%	1 (0)	0.52%	9 (3)	1.75%	179	0.50%	**0.016**	0.340	**0.011**
	26.6	*O*-methyl transferases	3 (3)	0.43%	3 (3)	1.57%	0	0	20	0.06%	**0.016**	**1.66E–04**	0.354
	8	TCA	6 (6)	0.85%	6 (6)	3.14%	0	0	87	0.24%	**0.016**	**6.39E–05**	0.778
	26.3	Gluco-, galacto- and mannosidases	5 (4)	0.71%	1 (1)	0.52%	4 (3)	0.78%	71	0.20%	**0.022**	0.131	**0.039**
	5	Fermentation	3 (3)	0.43%	0	0	3 (3)	0.58%	32	0.09%	**0.025**	0.266	**0.022**
	31.3	Cell cycle	7 (4)	0.99%	1 (1)	0.52%	6 (3)	1.17%	130	0.37%	**0.028**	0.263	**0.039**
	na	SUN-like proteins	3 (2)	0.44%	1 (1)	0.54%	2 (1)	0.40%	34	0.10%	**0.028**	0.055	0.060
	21.2	Ascorbate and glutathione	4 (2)	0.57%	2 (1)	1.05%	2 (1)	0.39%	61	0.17%	**0.038**	**0.027**	0.144
	24	Biodegradation of xenobiotics	4 (2)	0.57%	3 (1)	1.57%	1 (1)	0.19%	64	0.18%	**0.042**	**6.02E-03**	0.346
	21.1	Thioredoxin	4 (4)	0.57%	0	0	4 (4)	0.78%	75	0.21%	0.060	0.423	**0.039**
	26.28	GDSL-motif lipase	5 (5)	0.71%	4 (4)	2.09%	1 (1)	0.19%	112	0.32%	0.072	**6.02E–03**	0.571
	19	Tetrapyrrole synthesis	3 (2)	0.43%	3 (2)	1.57%	0	0	58	0.16%	0.075	**6.02E–03**	0.641
	26.2	UDP glucosyl and glucoronyl transferases	10 (10)	1.42%	7 (7)	3.66%	3 (3)	0.58%	338	0.95%	0.149	**7.03E–03**	0.778

*P*-values in bold indicate significant enrichment. a, All 687 differentially expressed genes between wild-type tomato and *MIR167* transgenic lines. b, The 185 genes that were differentially expressed both owing to overexpression of *MIR167* in tomato and in the *Arabidopsis arf6 arf8* double mutant. c, The 502 differentially expressed genes unique in tomato. d, Number of genes in the GO category in the entire tomato genome. e, Numbers in the parentheses are the numbers of down-regulated genes. f, Hypergeometric tests were performed in R using ‘phyper’. FDR method was used for *P*-value correction.

## Discussion

*ARF6* and *ARF8* orthologues are found in diverse angiosperms, including monocots and dicots (Remington *et al.*, 2004), but their roles in plant development have only been tested in *Arabidopsis* (Nagpal *et al.*, 2005; Reeves *et al.*, 2012; Ru *et al.*, 2006; Tabata *et al.*, 2010). In this study, the functions of the tomato *ARF6* and *ARF8* genes were evaluated by transgenically overexpressing the *Arabidopsis MIR167a* in this Solanaceous species. Although this approach does not distinguish between the functions of *ARF6* and *ARF8*, these genes have overlapping expression patterns and it seems likely that they act, at least partially, redundantly in tomato as in *Arabidopsis*.

Tomato plants overexpressing *MIR167a* had significantly decreased expression of *SpARF6A* and *SpARF8B*, and the flowers were arrested at the time of flower opening. The petals, stamens and styles were each shorter in mature post-anthesis flowers of *MIR167a* plants compared with wild type. Moreover, the style lacked trichomes that are normally present, and did not support wild-type pollen tube germination and/or growth resulting in female sterility. These phenotypes are similar to those described for flowers of *Arabidopsis arf6 arf8* mutants, which also arrest at the time of flower opening, with defects in petal, stamen, ovary, style, and stigma growth, and severely decreased ability of the gynoecium to support pollen tube growth (Crawford and Yanofsky, 2011; Nagpal *et al.*, 2005; Reeves *et al.*, 2012; Ru *et al.*, 2006; Tabata *et al.*, 2010). In each plant species, the organs that grow most rapidly at the time of flower opening are most strongly affected by decreasing ARF6 and ARF8: petals and the style in tomato, and petals and stamens in *Arabidopsis*.

*MIR167a* transgenic tomato plants also had shorter internodes and smaller leaves than did wild-type plants, indicating that the products of *SpARF6A* and *SpARF8B* genes play a role in promoting stem elongation and leaf expansion. *Arabidopsis arf6 arf8* mutants similarly have very short inflorescence stems, whereas they have only a subtle leaf phenotype. In *Arabidopsis*, ARF6 and ARF8 contribute to leaf expansion together with NPH4/ARF7 and ARF19 (Wilmoth *et al.*, 2005; JWR, unpublished results). In our RNA seq data set, overexpression of *MIR167a* led to increased expression of tomato *SpARF7/ARF19*, suggesting that it may compensate for the reduction in *SpARF6* and *SpARF8* function and perhaps have a similar role in regulating leaf expansion as in *Arabidopsis*.

Although our data demonstrate that *ARF6* and *ARF8* regulate flower maturation in *Arabidopsis* and tomato, differences between *MIR167a* transgenic tomato and *Arabidopsis arf6 arf8* phenotypes may reflect developmental or regulatory differences between the two species. In particular, the anthers of *MIR167a* tomato flowers still produced viable pollen, whereas the *Arabidopsis* mutants did not and were therefore male-sterile. This suggests either that the threshold for ARF6 and ARF8 action in tomato anthers is lower than was achieved by the *MIR167a* transgene expression, or that ARF6 and ARF8 do not regulate anther dehiscence in tomato.

The regulation of *ARF6* and *ARF8* by *miR167* is likely to be highly conserved. *miR167* is found in seed-producing plants from gymnosperms to flowering plants (Axtell and Bartel, 2005). In addition to targeting *ARF6* and *ARF8*, it was recently shown that *miR167* also guides cleavage of *IAA-Ala Resistant 3* (*IAR3*) transcripts in *Arabidopsis* (Kinoshita *et al.*, 2012). By using the SoMART degradome RNA library analysis, we demonstrate that *miR167* guides the cleavage of *SlARF6A*, *SlARF8A*, and *SlARF8B* transcripts in cultivated tomato, consistent with a previous report (Moxon *et al.*, 2008). We attempted to find and validate additional targets of *miR167* in tomato, which are listed in the miSolRNA database (www.misolrna.org). In addition to *SlARF6A* and *SlARF8A/B*, three putative candidate genes (Solyc04g077220, Solyc04g073990, and Solyc11g011980) could be targeted by *miR167*. However, none of them was validated as a likely target of *miR167* in the SoMART degradome RNA library database. Similarly, we could not identify breakdown products of the tomato orthologue of *IAR3* (Solyc03g121270) in the degradome RNA library database. It should be noted, however, that the SoMART degradome RNA library and other tomato degradome RNA libraries used leaf and fruit samples, but not root samples (Karlova *et al.*, 2013). *IAR3* is important for lateral root growth and thus, further studies on tomato root samples may help clarify whether *miR167* could mediate regulation of tomato *IAR3*. Thus far, there is no evidence that *miR167* in tomato regulates guided cleavage of any gene other than *ARF6A*, *ARF8A*, and *ARF8B*, consistent with findings from another study (Karlova *et al.*, 2013).

The phenotypes described above indicate that orthologous *ARF6* and *ARF8* genes have similar developmental functions in tomato and *Arabidopsis*. It will be interesting in future work to determine whether this similarity extends to targets of ARF regulation. The 187 putative orthologues whose expression is similarly altered in *arf6 arf8 Arabidopsis* mutants and *MIR167a* tomato (even though we examined distinct developmental stages using different experimental platforms) suggest that some targets may be conserved. Among these is *Style2.1* (which promotes style elongation) in tomato *MIR167a* plants, and the related *AtPRE1* gene in *Arabidopsis arf6 arf8* plants, which are underexpressed in *MIR167a* or *arf6 arf8* flowers (Chen *et al.*, 2007; Reeves *et al.*, 2012). Moreover, in both cases, some of the phenotypes could be attributed to reduced jasmonate production or signalling. In *Arabidopsis*, jasmonate is required for petal growth and anther dehiscence, and decreased jasmonate production accounts for a subset of *arf6 arf8* mutant phenotypes (Nagpal *et al.*, 2005; Reeves *et al.*, 2012; Tabata *et al.*, 2010). Analogously, tomato jasmonate-insensitive plants have defective stylar trichomes (Li *et al.*, 2004). Although we could not rescue the stylar development defect in tomato *MIR167a* plants with exogenous methyl jasmonate, decreased jasmonate production or response might nevertheless contribute to this aspect of the *MIR167a* overexpression phenotype. Similarly, a family of closely related *MYB* genes regulates various aspects of flower growth in *Arabidopsis*, ornamental tobacco, and Petunia (Cheng *et al.*, 2009; Colquhoun *et al.*, 2011; Liu *et al.*, 2009; Liu and Thornburg, 2012; Mandaokar *et al.*, 2006; Reeves *et al.*, 2012; Spitzer-Rimon *et al.*, 2010). These are underexpressed in *Arabidopsis arf6 arf8* flowers, and it will be interesting to determine whether members of this gene family in tomato similarly regulate flower growth downstream of *ARF6* and *ARF8*.

## Supplementary data

Supplementary data are available at *JXB* online

Figure S1. Mapped *Sly-miR167*-mediated cleaved *SlARF6A/SlARF8A/SlARF8B* RNAs and the predicted folding of the *Sly-miR167* genes.

Figure S2. Southern blot analysis of *MIR167* transgenic lines.

Figure S3. Pollen tube growth and gynoecium structure in *MIR167* and the wild-type LA1589.

Table S1. RPKM value for each gene in each replicate in 11 LA1589 tissues.

Table S2. Read count for *SpARF6A*, *SpARF6B*, *SpARF8A*, *SpARF8B* using the confirmed gene sequences.

Table S3. Floral organ lengths of *MIR167* and LA1589 control flowers.

Table S4. Differentially expressed genes in *MIR167* lines compared with LA1589 control in two comparisons.

Table S5. AuxRE Promoter analysis of the differentially expressed tomato genes

Table S6. Common differentially expressed genes in tomato and *Arabidopsis*.

Supplementary Data

## Abbreviations:

ARF: auxin response factor
Aux/IAA: auxin/indoleacetic acid protein
CTD: C-terminal dimerization domain
DBD: DNA-binding domain
DPA: days post-anthesis
GA: gibberellic acid
JA: jasmonic acid
MeJA: methyl jasmonic acid
MIR: microRNA precursor gene.
