# Supplementary material for: Down-regulation of AUXIN RESPONSE FACTORS 6 and 8 by microRNA 167 leads to floral development defects and female sterility in tomato
Source: J Exp Bot. 2014 Apr 10;65(9):2507–20. doi: 10.1093/jxb/eru141 (PMC4036516; doi:10.1093/jxb/eru141)
Supplement: Supplementary Data [file supp_65_9_2507__index.html]

Down-regulation of AUXIN RESPONSE FACTORS 6 and 8 by microRNA 167 leads to floral development defects and female sterility in tomato — Down-regulation of AUXIN RESPONSE FACTORS 6 and 8 by microRNA 167 leads to floral development defects and female sterility in tomato — Supplementary Data 

# Down-regulation of *AUXIN RESPONSE FACTORS 6* and *8* by microRNA 167 leads to floral development defects and female sterility in tomato

## Supplementary Data

Data files

**Files in this Data Supplement:**

- Supplementary Data - Supplementary Data
- Supplementary Data - Supplementary Data
