# Supplementary material for: Down-regulation of AUXIN RESPONSE FACTORS 6 and 8 by microRNA 167 leads to floral development defects and female sterility in tomato
Source: J Exp Bot. 2014 Apr 10;65(9):2507–20. doi: 10.1093/jxb/eru141 (PMC4036516; doi:10.1093/jxb/eru141)

Supplemental figures for “Downregulation of *AUXIN RESPONSE FACTORS 6* and *8* by microRNA 167 leads to floral development defects and female sterility in tomato”

Authors: Ning Liu, Shan Wu, Jason Van Houten, Ying Wang, Biao Ding, Zhangjun Fei, Thomas H. Clarke, Jason W. Reed, Esther van der Knaap

Figure S1. Mapped *Sly-miR167*-mediated cleaved *SlARF6A/SlARF8A/SlARF8B* RNAs and the predicted folding of the *Sly-miR167* RNAs. A. Mapped cleavage sites of *SlARF6A*, *SlARF8A*, and *SlARF8B* RNAs identified by the SoMART software package. For each target transcript, shown are the base-pairing with *miR167*, the position of the target cleavage sites, and the read numbers of the matching RNA fragments in the degradome library. The cleavage sites are highlighted by arrows. "|" indicates canonical base pairing and "o" indicates G:U wobble pairing. **B.** The predicted folding of stem-loop structures in *Sly-MIR167a-1*, *Sly-MIR167a-2*, *Sly-MIR167a-3*, and *Sly-MIR167a-4*. The genomic locations of precursor sequences are given in parentheses. The folding was predicted by mFOLD (hUUp://mfold.rna.albany.edu). The mature *miR167* sequence is highlighted in bold green. **C.** Two *miR167a-like* sequences that could not be validated as miRNAs. The alignment of *miR167a-like* and *miR167a* sequences are shown with differing nucleotides highlighted in pink shade in *miR167a-like* sequences. The in silico transcribed genomic sequences are shown with predicted RNA folding, with the *miR167a-like* sequences highlighted in green. Note: by using the data from http://smallrna.udel.edu/, we found 6 reads in both leaf and floral samples for *miR167a-like-1* and 1 read in floral sample for *miR167a-like-2*. No reads for the predicted *miR167a-like** were found.

A, Sly-miR167 mediates mRNA cleavage of SlARF6A, SlARF8A, and SlARF8B.

SlARF6A

```
                     10/10
                        ↓
2365 5'-CCUGAGAGAUCAGGCUGGCAGCUUGUAUUCGU-3' 2396 cleavage site: 2381
              |||||| ||||||||||| o|
          3'-AUCUAGUACGACCGUCGAA-GU-5'
```

```
5′-uccugagagaucaggcuggcagcuuguauucguugaccgugagaaugauguucuccuccuugguggaugaccccuggcaugaguuug-3′
                   ggcagcuuguauucguugaccgugagaaugauguucuccuccuugguggaugaccccuggcaugaguuugug (7 reads)
                   ggcagcuuguauucguugaccgugagaaugauguucuccuccuugguggaugaccccugg             (3 reads)
```

SlARF8A

```
                   2/16 14/16
                       ↓↓
2278 5'-CCUCAAAGAUCAGGCUGGCAGCUUGUAUUUGU-3' 2309 cleavage site: 2294
              |||||| ||||||||||| o|
          3'-AUCUAGUACGACCGUCGAA-GU-5'
```

```
5′-cccucaaagaucaggcuggcagcuuguauuuguugacagggagaaugauguccuucuccuuggagacgauccgugggaggaauuug-3′
                   ggcagcuuguauuuguugacagggagaaugauguccuucuccuuggagacgauccgugggaggaauuuguc (2 reads)
                  uggcagcuuguauuuguugacagggagaaugauauccuucuccuuggagacgaucc                (1 reads)
                   ggcagcuuguauuuguugacagggagaaugauauccuucuccuuggagacgauc                 (2 reads)
                   ggcagcuuguauuuguugacagggagaaugauauccuucuccuuggagacgau                  (1 reads)
                   ggcagcuuguauuuguugacagggagaaugauauccuucuccuuggagacg                    (4 reads)
                   ggcagcuuguauuuguugacagggagaaugauauccuucuccuuggaga                      (1 reads)
                   ggcagcuuguauuuguugacagggagaaugauaucc                                   (1 reads)
                   ggcagcuuguauuuguugacagggagaaugauguucc                                  (1 reads)
                   ggcagcuuguauuuguugacagggag                                             (1 reads)
                  uggcagcuuguauuuguugac                                                   (1 reads)
                   ggcagcuuguauuuguuga                                                    (1 reads)
```

SlARF8B

```
                   2/64 62/64
                       ↓↓
```

```
2275 5'-CCUCAAAGAUCAGGCUGGCAGCUUGUAUUUGU-3' 2306 cleavage site: 2291
              |||||| ||||||||||| o|
          3'-AUCUAGUACGACCGUCGAA-GU-5'
```

```
5′-ccucaaagaucaggcuggcagcuuguauuuguugacagggagaaugauauccuucuccuuggagacgauccaugggaggcguuuguc-3′
                   ggcagcuuguauuuguugacagggagaaugauauccuucuccuuggagacgauccaugggaggcguuuguc  (23reads)
                   ggcagcuuguauuuguugacagggagaaugauauccuucuccuuggagacgauccaugggaggcguuug    (3 reads)
                   ggcagcuuguauuuguugacagggagaaugauauccuucuccuuggagacgauccaugggaggcguuu     (5 reads)
                   ggcagcuuguauuuguugacagggagaaugauauccuucuccuuggagacgauccaugggaggc         (4 reads)
                   ggcagcuuguauuuguugacagggagaaugauauccuucuccuuggagacgauccaugggagg          (1 reads)
                   ggcagcuuguauuuguugacagggagaaugauauccuucuccuuggagacgauccaugggag           (2 reads)
                   ggcagcuuguauuuguugacagggagaaugauauccuucuccuuggagacgauccauggg             (4 reads)
                   ggcagcuuguauuuguugacagggagaaugauauccuucuccuuggagacgauccau                (3 reads)
                   ggcagcuuguauuuguugacagggagaaugauauccuucuccuuggagacgaucca                 (3 reads)
                  uggcagcuuguauuuguugacagggagaaugauauccuucuccuuggagacgaucc                  (1 reads)
                   ggcagcuuguauuuguugacagggagaaugauauccuucuccuuggagacgauc                   (2 reads)
                   ggcagcuuguauuuguugacagggagaaugauauccuucuccuuggagacgau                    (1 reads)
                   ggcagcuuguauuuguugacagggagaaugauauccuucuccuuggagacg                      (4 reads)
                   ggcagcuuguauuuguugacagggagaaugauauccuucuccuuggaga                        (1 reads)
                   ggcagcuuguauuuguugacagggagaaugauaucc                                     (1 reads)
                   ggcagcuuguauuuguugacagggagaaugauguccc                                    (1 reads)
                   ggcagcuuguauuuguugacagggagaaugauau                                       (2 reads)
                   ggcagcuuguauuuguugacagggag                                               (1 reads)
                  uggcagcuuguauuuguugac                                                     (1 reads)
                   ggcagcuuguauuuguuga                                                      (1 reads)
```

B. Predicted precursor structures of *Sly-miR167a* genes

Sly-MIR167a-1 **(SL2.40ch06:45629365..45629463-)**

```
      CUA    U   A       G        AAA        UU    CAA
5′- CA   GCAG UGA GCUGCCA CAUGAUCU   CUUUCCUU  AGUU   A
3′- GU   UGUC AUU CGACGGU-GUACUAGA   GAAAGGAG  UUAA   U
      AAC    C   C                CUA        -C    UA-
```

Sly-MIR167a-2 **(SL2.40ch09:59575900..59576000+)**

```
     U    CA      A                    A     C       AAA
5′- G GGAA  AGUGA GCUG-CC-AGCAUGAUCUAA CUUGG CAUUUAU   G
3′- U CCUU  UCACU CGAC GG UCGUACUGGAUU GAAUC GUGGGUA   A
     U    CC      C    A  C            G     A       -AA
```

Sly-MIR167a-3 **(SL2.40ch09:59584678..59584769+)**

```
      C      U   A       G        AAA       AU   U
5′- CA UAGCAG UGA GCUGCCA CAUGAUCU   CUUUUCU  UGC C
3′- GU GUUGUC ACU CGACGGU-GUACUAGA   GAAAGGA  AUG C
      A      C   A                CUA       GC   -
```

Sly-MIR167a-4 **(SL2.40ch09:63883405..63883497+)**

```
    U    U   U      U    G        AAA   A   A   AUU
```

```
5′-  ACUA CAG UGAAGC GCCA CAUGAUCU   CUU CCU UAA   U
3′-  UGAU GUC ACUUCG UGGU-GUACUAGA   GAA GGA-AUU   A
    U    U   C      U             CUA   C       AU-
```

C. *miR167a-like* sequences

miR167-like-1

```
     miR167a: 5′- UGAA-GCUGCCAGCAUGAUCUA -3′
miR167a-like: 5′- UUAAAGCUGCCAGCAUGAUCUG -3′
```

```
    UC   UCUG         U-      G
5′-   ACU    UAUGUUGGU  UUUUGA A
3′-   UGG    GUACGACCG  GAAAUU G
     A   UCUA         UC      G
```

miR167-like-2

```
     miR167a: 5′- UGAAGCUGCCAGCAUGAUCUA -3′
miR167a-like: 5′- UGAAGAUGCCAGCAUGAUCU -3′
```

```
                    UG
                   A  A
                   C  U
                    GC
                    AU
                    CG
                    CG
                    GU
    G       U-     U  GACAACAUACA
5′-  AG-AGGU  GAAGA              U
3′-  UC UUCA  CUUUU-  AUAUAUAUACA
    U  U    UC      UA
                    UA
                    CG
                    GC
                    UA
                    UA
                    UA
                   A  A
                    AG
```

Figure S2. Southern blot analysis of *MIR167* transgenic lines. Genomic DNA was digested with *Eco*RI or *Eco*RV, and separated on 0.8% agarose gels. Non-transgenic LA1589 served as control. The *BAR* gene was used as probe.

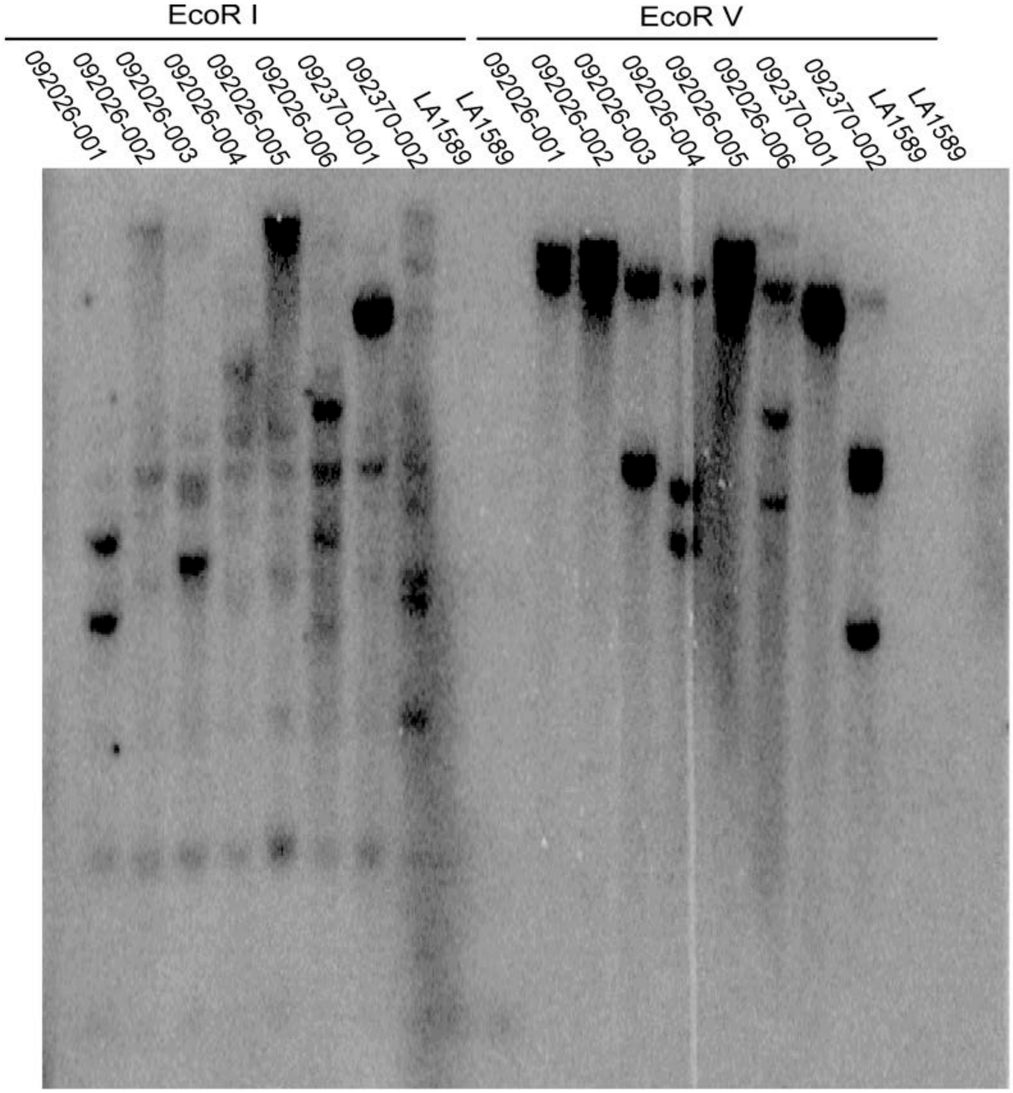

Figure S3. Pollen tube growth and gynoecium structure in *MIR167* and control tomato. **A - B.** Control LA1589 pistils pollinated with *MIR167* pollen from severe T_0 lines 092026-003 and 092026-004, respectively. **C.** Two *MIR167* pistils pollinated with LA1589 pollen. Pistils were collected 16 hours after pollination, and pollen tubes were visualized by aniline blue staining. Arrows indicate pollen tubes and arrowheads indicate the vascular bundle. **A-C** Scale bar = 0.5 mm. **D – G.** Cross-section of the style at the middle position 1 day before anthesis. **H – K.** Cross-section of the style at the position proximal to the ovary at 1 day before anthesis. **L – M.** Cross-section of the ovary. Wild type (**D, H, L**); T_0 line without phenotype 090226-005 (**E, I**); severe T_0 lines 092026-003 (**F, J, M**) and 092026-004 (**G, K**).

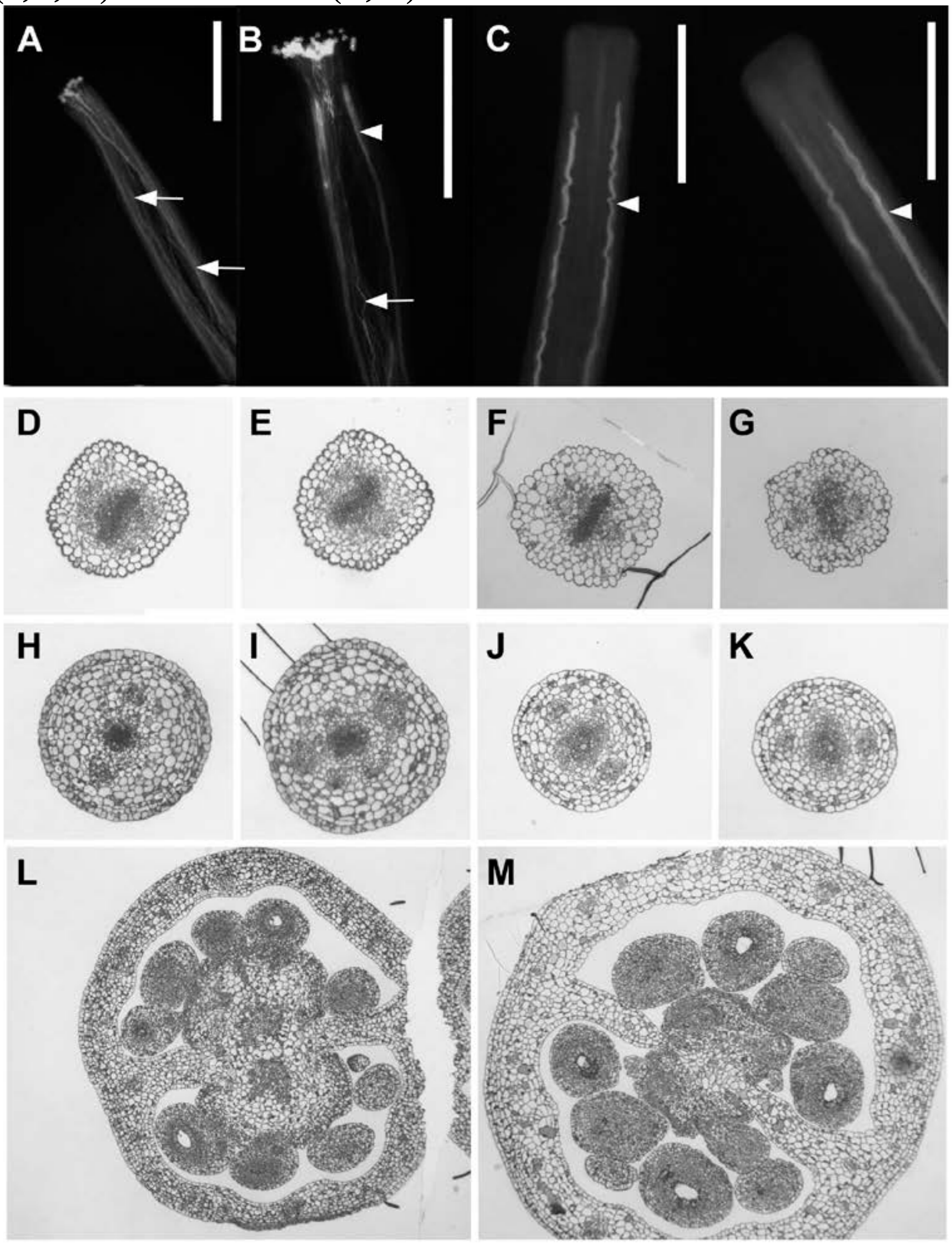

Supplement: Supplementary Data [file supp_eru141_jexbot116111_file001.pdf]
